# Carotid Artery Stenosis: Comparison of 3D Time-of-Flight MR Angiography and Contrast-Enhanced MR Angiography at 3T

**DOI:** 10.1155/2014/508715

**Published:** 2014-03-23

**Authors:** Ivan Platzek, Dominik Sieron, Philipp Wiggermann, Michael Laniado

**Affiliations:** ^1^Department of Radiology, Dresden University Hospital, Fetscherstraße 74, 01307 Dresden, Germany; ^2^Department of Radiology, District Hospital of Orthopedics and Trauma Surgery, Ulica Bytomska 62, 41-941 Piekary Śląskie, Poland; ^3^Department of Radiology, Regensburg University Medical Center, Franz-Joseph-Strauß-Allee 11, 93053 Regensburg, Germany

## Abstract

*Purpose*. The aim of this study was to assess the correlation of 3D time-of-flight MR angiography (TOF MRA) and contrast-enhanced MR angiography (CEMRA) for carotid artery stenosis evaluation at 3T. *Material and Methods*. Twenty-three patients (5 f, 18 m; mean age 61 y, age range 45–78 y) with internal carotid artery stenosis detected with ultrasonography were examined on a 3.0T MR system. The MR examination included both 3D TOF MRA and CEMRA of the carotid arteries. MR images were evaluated independently by two board-certified radiologists. Stenosis evaluation was based on a five-point scale. Stenosis grades determined by TOF and CEMRA were compared using Spearman's rank correlation coefficient and the Wilcoxon test. Cohen's Kappa was used to evaluate interrater reliability. *Results*. CEMRA detected stenosis in 24 (52%) of 46 carotids evaluated, while TOF detected stenosis in 27 (59%) of 46 carotids. TOF MRA yielded significantly higher results for stenosis grade in comparison to CEMRA (*P* = 0.014). Interrater agreement was very good for both TOF MRA (*κ* = 0.93) and CEMRA (*κ* = 0.93). *Conclusion*. At 3T, 3D TOF MRA should not be used as replacement for contrast-enhanced MRA of the carotid arteries, as it results in significantly higher stenosis grades.

## 1. Introduction

Atherosclerotic disease of the carotid arteries has a high prevalence in patients aged over 50 and is a major cause of ischemic stroke [1, 2]. While digital subtraction angiography (DSA) is still viewed as the gold standard in carotid imaging, noninvasive imaging methods, including resonance angiography (MRA), computed tomography angiography (CTA), and ultrasonography, play an increasing role in the evaluation of carotid artery disease. In addition to being noninvasive, MRA does not utilize ionizing radiation. Currently MRA of the extracranial carotid arteries is mostly performed as contrast-enhanced angiography (CEMRA), after intravenous injection of gadolinium-based contrast agents. While MR contrast agents have very few side effects [3, 4], they may cause nephrogenic systemic fibrosis (NSF) in patients with renal insufficiency [5]. The awareness of this possible side effect of gadolinium-based contrast agents has led to increased interest in nonenhanced MRA in recent years [6]. The most widely used type of nonenhanced MRA is time-of-flight angiography (TOF). TOF has been routinely used for imaging the intracranial arteries for many years [7]. Due to improved hardware, TOF with good spatial resolution is now feasible for carotid artery imaging in a reasonable timeframe. While most MR examinations are still performed on 1.5T scanners, the use of 3T systems in clinical routine is rising. 3T systems offer a higher signal-to-noise ratio, which can help improve image quality without an increase in scan time.

The purpose of this study was to assess the agreement between TOF and CEMRA in carotid artery stenosis evaluation at 3T.

## 2. Materials and Methods

This prospective study was approved by the local ethics committee and written informed consent was obtained from all patients. Twenty-three patients (5 f, 18 m; mean age 61 y, age range 45–78 y) with external carotid artery stenosis diagnosed with ultrasonography were included in the study. One patient was excluded because the MR examination could not be completed due to claustrophobia.

Fifteen patients had no neurological symptoms. Three patients had amaurosis fugax, two patients had transient ischemic attack (TIA), one patient had paresthesia and weakness of the left arm, one patient had syncope, and one had facial nerve paralysis. Three patients had a history of previous stroke.

### 2.1. MR Angiography

All patients were examined on a 3T MR scanner (Magnetom Trio, Siemens Medical Solutions, Erlangen, Germany). The patients were examined in the supine position. An 18G intravenous catheter was placed in a vein of the right antecubital fossa before the examination and was later used for contrast injection. The vendor-supplied head and neck coils were used for signal acquisition.

The extracranial carotid arteries were examined with both TOF MRA and CEMRA, with TOF MRA being performed first.

3D TOF MRA was acquired in transverse orientation and covered the carotid bifurcation and the adjacent parts of the extracranial carotid arteries. It was performed with 25 ms/3.1 ms (repetition time/echo time), 74 slices, 0.8 mm slice thickness, a field of view of 149 × 199 mm, and a matrix of 288 × 384. The 3D TOF slab was acquired in multislab technique (three 3D slabs of equal thickness). The 3D TOF MRA protocol utilizes TONE (tilted optimized nonsaturating excitation) to reduce saturation effects [7]; the mean flip angle was 25°. A saturation band placed superior to the acquisition volume was used to eliminate venous flow signal. Acquisition time was 05 : 01 min.

A test bolus timing scan was performed before the acquisition of the contrast-enhanced MR angiography in order to determine the contrast agent arrival time. The test bolus consisted of 2 mL 0.5 M gadopentetate dimeglumine (Magnevist, Bayer Schering Pharma, Berlin, Germany), injected with 2 mL/s, followed by 20 mL of saline. An MR compatible dual head injector (Spectris Solaris, Medrad, Indianola, PA, USA) was used for injection of both the contrast agent and the saline flush. The test bolus timing scan is a dynamic 2D gradient echo scan, which consists of 60 consecutive acquisitions of a single slice in identical position. The test bolus scan was performed with 12 ms/1.1 ms (repetition time/echo time), flip angle 20°, 1 slice, 12 mm slice thickness, a field-of-view of 20 × 20 cm, and a matrix of 128 × 128. The test bolus timing scan was acquired in transverse orientation just inferior to the carotid artery bifurcation and was started simultaneously with the test bolus injection.

The test bolus scan was used to generate a time intensity curve, which allows determining the time to peak enhancement [8] and deciding when to start the acquisition of the contrast-enhanced diagnostic MRA after applying the full bolus. The time intensity curve was calculated by the scanner's software package (Syngo MR B13, Siemens Medical Solutions, Erlangen, Germany) after a region of interest (ROI) was placed in the left common carotid artery by the radiation technologist. The scan delay was calculated by subtracting 6.2 s from the arterial time to peak enhancement, which is determined using the time intensity curve. 6.2 s are the time when the 3D sequence reaches its k-space center. This value is provided by the vendor.

Contrast-enhanced MR angiography was performed in coronal orientation with 2.7 ms/1.1 ms (repetition time/echo time), flip angle 25°, 88 slices, 0.7 mm slice thickness, a field-of-view of 343 × 500 mm, and a matrix of 352 × 512, with sequential k-space ordering. The contrast-enhanced MR angiography is performed in subtraction technique, which means that datasets with identical parameters covering the same region are performed before and after contrast injection and the resulting magnitude images are then subtracted. Acquisition time was 21 s for both the contrast-enhanced and nonenhanced datasets.

The contrast-enhanced scan was acquired after intravenous injection of 20 mL Magnevist. The injection rate was 2 mL/s. The contrast agent was followed by a 20 mL flush of 0,9% saline, also injected with 2 mL/s. The same dual head injector already described above was used.

### 2.2. Image Evaluation

MR images were evaluated independently by two board-certified radiologists (with eight-year and four-year experience with MR angiography, resp.). Both the 3D TOF and 3D CEMRA primary datasets and maximum intensity projection (MIP) images were reviewed. Stenosis grade was primarily determined based on multiplanar reconstructions of the 3D datasets. Image evaluation was performed on a LEONARDO postprocessing workstation with the Syngo MR A35 software (Siemens Medical Solutions, Erlangen, Germany).

TOF MRA and CEMRA were evaluated separately, with a four-week time interval between evaluation sessions. While evaluating TOF MRA, the readers were blinded for CEMRA images and vice versa. Furthermore, the readers were blinded for other imaging or clinical data. In cases of interrater differences concerning the same MR angiography type, stenosis grade was determined by the readers in consensus.

Stenosis evaluation was based on a five-point scale: 0 = normal; 1 = mild stenosis, less than 50%; 2 = moderate stenosis, 50–69%; 3 = severe stenosis, more than 70% but less than full occlusion; 4 = occlusion.

### 2.3. Statistical Analysis

Stenosis grades determined by TOF and CEMRA were compared using the Spearman rank correlation coefficient and the Wilcoxon test. A *P* value ≤ 0.05 was considered statistically significant. Cohen's Kappa was used to evaluate interrater reliability. Data were analyzed using MedCalc 12.5 (MedCalc Software, Ostend, Belgium).

## 3. Results

CEMRA detected stenosis in 24 (52%) of 46 carotids evaluated (occlusion: *n* = 5; severe stenosis: *n* = 11; moderate stenosis: *n* = 5; mild stenosis: *n* = 3).

**Figure 1:**
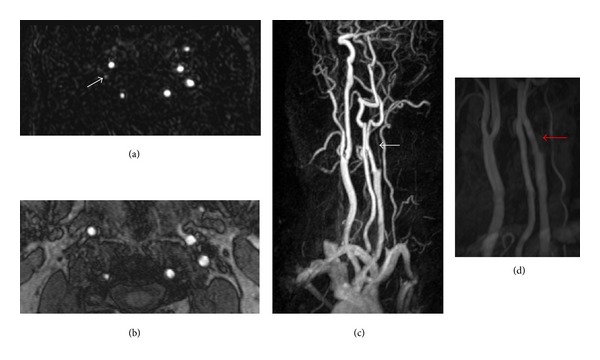
Forty-nine-year-old male patient with high-grade asymptomatic stenosis of the right external carotid artery detected with ultrasonography. While a filiform stenosis is seen on the contrast enhanced images (white arrow), the lumen of the right internal carotid artery is not seen on TOF images and the finding was interpreted as occlusion (red arrow). (a) Axial reformat of the subtracted contrast-enhanced MR angiography (CEMRA), (b) maximum intensity projection (MIP) of the CEMRA dataset, (c) axial time-of-flight (TOF) MR angiography image, and (d) MIP of the TOF MRA dataset. The much smaller axial field-of-view of the TOF MRA is clearly seen on the MIP images.

In contrast, TOF detected stenosis in 27 (59%) of 46 carotids (occlusion: *n* = 8; severe stenosis: *n* = 12; moderate stenosis: *n* = 5; mild stenosis: *n* = 2) (Figure 1).

On both CEMRA and TOF, unilateral stenosis was found in 16 (69%) of 23 patients and bilateral stenosis in five (22%) of 23 patients, while no stenosis was detected in two (8.7%) of 23 patients. There were no cases with tandem stenoses and no evidence of ulcerated plaque.

Spearman's rank correlation coefficient was calculated to be *ρ* = 0.96. TOF MRA yielded significantly higher results for stenosis grade in comparison to CEMRA (*P* = 0.014).

Stenosis evaluation results are summarized in Table 1.

When evaluating TOF images, the readers agreed on stenosis grade in 40 (87%) of 46 cases and had differing results in 6 (13%) of 46 cases. For CEMRA evaluation, the readers had identical results for stenosis grade in 40 (87%) of 46 cases and differing results in 6 (13%) of 46 cases. Interrater agreement was very good for both TOF MRA (*κ* = 0.93) and CEMRA (*κ* = 0.93).

## 4. Discussion

Our study shows that the use of 3D TOF MRA results in significantly higher stenosis grades compared to CEMRA when evaluating carotid arteries at 3T. Our results also indicate good rank correlation between TOF MRA and CEMRA, but given the first finding this is only of secondary importance. Good rank correlation only means that a stenosis which received a high score with one method also received a relatively high score with the second method, while it does not describe agreement between individual scores.

While our study did not include a comparison with a standard of reference like DSA or rotational angiography, the higher stenosis grades yielded by TOF MRA in comparison to CEMRA at 3T represent a problem, as CEMRA of the carotid arteries is well validated. CEMRA has been shown to have a high sensitivity and specificity for extracranial carotid artery stenosis at both 1.5T [9–11] and 3T [12]. Anzalone et al. were also able to show that CEMRA of the carotid arteries correlates better with rotational angiography than DSA [13]. Thus the use of TOF MRA is not recommended for evaluation of the extracranial carotid arteries at 3T at this stage because of significant lack of agreement with the well-validated CEMRA.

To our knowledge there are no previous studies about the use of 3D TOF MRA for carotid stenosis evaluation at 3T. Our results show parallels to the study of Kang et al., who compared 2D TOF and CEMRA for stenosis evaluation of peripheral, renal, and carotid arteries at 3T [14]. Kang et al. found CEMRA to have better sensitivity and specificity for carotid artery stenosis than TOF. An important difference is the use of 3D TOF in the current study, instead of 2D TOF used by Kang et al., with a better spatial resolution (0.8 mm slice thickness and 288 × 384 matrix versus 1.5 mm slice thickness and 256 × 256 matrix).

The use of TOF MRA of the extracranial carotid arteries at 1.5T has been studied extensively, however, with partially conflicting results. Anzalone et al. [13] and Scarabino et al. [15] found no significant difference between TOF and CEMRA regarding sensitivity and specificity for carotid artery stenosis at 1.5T. In contrast, Fellner et al. found 3D TOF to be more accurate then CEMRA [16]. Townsend et al. showed CEMRA to overestimate the severity of carotid stenosis compared to 3D TOF, both at 1.5T [17]. One possible explanation for differences between the results of the current study and the studies mentioned above can be the improved spatial resolution of newer generation MR scanners, especially the improved spatial resolution of CEMRA. Another possible factor is bolus timing, which is crucial for CEMRA image quality. In the current study, we used a test bolus technique [18], which in general allows for a reliable assessment of delay time. Nevertheless, the selection of the proper delay time after the test bolus is dependent on the experience of the technologist and is a potential cause of errors. The results may also be influenced by the percentage of high-grade stenoses in our study population. Signal intensity saturation of low flow distal to high-grade stenoses is a known problem of TOF MRA [19] and may cause the readers to overestimate stenosis grade on TOF images especially in patients with high-grade stenosis. Furthermore, the influence of field strength on artifacts in TOF MRA has not been investigated yet.

The current study has several limitations. No digital subtraction angiography was performed in the patients included in the study and thus we were not able to determine sensitivity and specificity of both CEMRA and TOF. However, as discussed above, contrast-enhanced MRA of the carotid arteries is already well validated at both 1.5T and 3T. Another limitation is the large difference in axial field-of-view: 74 mm for TOF MRA versus 500 mm for CEMRA. The axial field-of-view of TOF MRA was restricted in order to reduce scan time, as scan time remains a significant disadvantage of TOF MRA. While all stenoses detected in this study are localized at or near the carotid bifurcation, the small axial field-of-view is a problem as it may cause tandem stenoses to be missed. The whole length of the extracranial carotid arteries can be depicted by TOF MRA, however, at the cost of even longer scan times. In analogy to previous comparable studies, we included both carotids of each patient in our evaluation, even if there was no clinical or sonographic evidence for bilateral stenosis. This approach may cause overrepresentation of vessels with no stenosis and artificially improve the agreement between TOF MRA and CEMRA.

## 5. Conclusions

In conclusion, 3D TOF MRA at 3T should not be used as replacement for contrast-enhanced MRA of the carotid arteries, as it results in significantly higher stenosis grades, and may potentially lead to inadequate therapy. Comparison with DSA and especially rotation angiography is needed for a more comprehensive assessment of the possibilities of TOF MRA at 3T.

## Figures and Tables

**Table 1:** Stenosis grade results.

Patient no.	Side	Stenosis grade TOF	Stenosis grade CEMRA
1	Right	4	4
	Left	0	0

2	Right	2	2
	Left	4	4

3	Right	3	2
	Left	2	1

4	Right	2	2
	Left	4	4

5	Right	0	0
	Left	3	2

6	Right	0	0
	Left	3	3

7	Right	1	1
	Left	1	1

8	Right	3	3
	Left	0	0

9	Right	3	3
	Left	0	0

10	Right	2	1
	Left	0	0

11	Right	4	3
	Left	0	0

12	Right	3	2
	Left	0	0

13	Right	3	2
	Left	2	3

14	Right	4	3
	Left	0	0

15	Right	0	0
	Left	4	3

16	Right	0	0
	Left	0	0

17	Right	0	0
	Left	4	4

18	Right	3	2
	Left	0	0

19	Right	0	0
	Left	0	0

20	Right	3	3
	Left	0	0

21	Right	0	0
	Left	3	3

22	Right	3	3
	Left	0	0

23	Right	3	3
	Left	0	0

TOF: time-of-flight MR angiography; CEMRA: contrast-enhanced MR angiography. 0 = normal; 1 = mild stenosis, less than 50%; 2 = moderate stenosis, 50–69%; 3 = severe stenosis, more than 70% but less than full occlusion; 4 = occlusion.
